# Identification of novel B-1 transitional progenitors by B-1 lymphocyte fate-mapping transgenic mouse model *Bhlhe41* ^dTomato-Cre^

**DOI:** 10.3389/fimmu.2022.946202

**Published:** 2022-09-15

**Authors:** Hui Li, Yangyang Tang, Jinfeng Ren, Ruixue Bai, Lang Hu, Wenyu Jia, Yiwei Cao, Li Hong, Meizhen Xu, Sijia Gao, Yanbiao Shi, Shuai Pan, Liang Wang, Kuiyang Zheng, Shuli Zhao, Hui Wang

**Affiliations:** ^1^ Jiangsu Key Laboratory of Immunity and Metabolism, Department of Pathogenic Biology and Immunology, Xuzhou Medical University, Xuzhou, China; ^2^ National Experimental Demonstration Center for Basic Medicine Education, Xuzhou Medical University, Xuzhou, China; ^3^ Department of Nursing, Jiangsu Provincial Xuzhou Pharmaceutical Vocational College, Xuzhou, China; ^4^ Department of Dermatology, The Affiliated Hospital of Xuzhou Medical University, Xuzhou, China; ^5^ Department of Biotechnology, School of Life Sciences, Xuzhou Medical University, Xuzhou, China; ^6^ Institute of Neuroscience and Department of Neurology of The Second Affiliated Hospital, National Health Commission and Chinese Academy of Medical Sciences Key Laboratory of Medical Neurobiology, Zhejiang University School of Medicine, Hangzhou, China; ^7^ General Clinical Research Center, Nanjing First Hospital, Nanjing Medical University, Nanjing, China

**Keywords:** B-1, B-1 progenitors, fate mapping, Bhlhe41, dTomato, EYFP

## Abstract

B-1 lymphocytes exhibit specialized roles in host defense against multiple pathogens. Despite the fact that CD19^+^CD93^+^B220^lo/-^ B cells have been identified as B-1 progenitors, the definition for B-1 progenitors remains to be elucidated as CD19^+^CD93^+^B220^+^ B cells are capable to give rise to B-1 cells. Given that transcription factor Bhlhe41 is highly and preferentially expressed in B-1 cells and regulates B-1a cell development, we generated a transgenic mouse model, *Bhlhe41^dTomato-Cre^*, for fate mapping and functional analysis of B-1 cells. *Bhlhe41^dTomato-Cre^* mice efficiently traced Bhlhe41 expression, which was mainly restricted to B-1 cells in B-cell lineage. We showed an efficient and specific Cre-mediated DNA recombination in adult B-1 cells and neonatal B-1 progenitors rather than B-2 cells by flow cytometric analysis of *Bhlhe41* ^dTomato-Cre/+^ *Rosa26* ^EYFP^ mice. Treatment of *Bhlhe41* ^dTomato-Cre/+^ *Rosa26* ^iDTR^ mice with diphtheria toxin revealed a robust efficacy of B-1 cell depletion. Interestingly, using *Bhlhe41* ^dTomato-Cre^ mice, we demonstrated that neonatal B-1 progenitors (CD19^+^CD93^+^B220^lo/-^) expressed Bhlhe41 and were identical to well-defined transitional B-1a progenitors (CD19^+^CD93^+^B220^lo/-^CD5^+^), which only gave rise to peritoneal B-1a cells. Moreover, we identified a novel population of neonatal splenic CD19^hi^dTomato^+^B220^hi^CD43^lo^CD5^lo^ B cells, which differentiated to peritoneal B-1a and B-1b cells. *Bhlhe41* deficiency impaired the balance between CD19^hi^dTomato^+^B220^lo/-^CD5^hi^ and CD19^hi^dTomato^+^B220^hi^CD5^lo^ cells. Hence, we identified neonatal CD19^hi^dTomato^+^B220^hi^CD43^lo^CD5^lo^ B cells as novel transitional B-1 progenitors. *Bhlhe41* ^dTomato-Cre/+^ mouse can be used for fate mapping and functional studies of B-1 cells in host-immune responses.

## Introduction

B lymphocytes are crucial immune cells in humoral immune responses by secreting different subtypes of antibodies in response to invading pathogens (1, 2). B lymphocytes can be classified as B-1 and B-2 cells, the latter of which include follicular B lymphocytes and marginal zone B lymphocytes (1, 2). Different from follicular B lymphocytes, which are key players in adaptive immunity and preferentially respond to thymus-dependent antigens, B-1 cells together with marginal zone B cells are more prone to respond to thymus-independent antigens, thereby contributing as innate effector cells in controlling invading pathogens prior to adaptive immune responses (1–3).

The B-2 cell development occurs at bone marrow and can be divided to sequential stages including progenitor B cells, pre–B cells, and immature B cells, the latter of which migrate to the spleen where B cells mature through transitional 1 (T1), T2, and T3 stages into follicular B cells and marginal zone B cells at secondary lymphoid organs (2). In contrast to B-2 cells that are replenished throughout life at the bone marrow, B-1 cells developed from the fetal liver followed by contribution from neonatal bone marrow and spleen (4–6). However, B-1 cell compartment cannot be fully reconstituted by bone marrow from adult mice (7). Instead, B-1 cells are self-renewed throughout life and generally reside in peritoneal and pleural cavities (1).

B-1 cells are divided as B-1a (CD5^+^) and B-1b cells (CD5^-^) according to surface CD5 expression. It has been established that B-1 progenitors are Lin^-^CD19^+^CD93^+^B220^lo/-^ and can be identified in the fetal liver, bone marrow, and spleen of neonatal and adult mice (8, 9). In addition, transitional B-1a progenitors (CD19^+^CD93^+^B220^lo/-^CD5^+^) that exclusively give rise to B-1a cells are identified in the neonatal spleen (10, 11). However, Lin^-^CD19^+^CD93^+^B220^+^ pro B cells can give rise to both B-2 and B-1 cells in peritoneal cavity (9, 12), indicating that B-1 progenitors may also present in Lin^-^CD19^+^CD93^+^B220^+^ population.

Multiple transgenic mouse models such as *Cd19^Cre^*, *Cd21^Cre^*, and *Mb1^Cre^* have been established for targeting B-2 cells at different stages of B-cell development. However, a transgenic mouse model for fate mapping and functional analysis of B-1 cells is still lacking so far. Transcription factor Bhlhe41 is recently reported to be preferentially expressed in B-1 cells and regulates B-1a cell development and self-renewal (10). In this study, we generated a transgenic mouse model, termed *Bhlhe41^dTomato-Cre^*, for fate mapping and functional analysis of B-1 cells. We showed an efficient and B-1 cell–specific Cre-recombinase–mediated DNA recombination in *Bhlhe41* ^dTomato-Cre/+^ *Rosa26* ^EYFP^ mice and a robust depletion of B-1 cells in *Bhlhe41* ^dTomato-Cre/+^ *Rosa26* ^iDTR^mice upon injection of diphtheria toxin. In addition, using *Bhlhe41* ^dTomato-Cre^ mice, we identified that neonatal B-1 progenitors (CD19^+^CD93^+^B220^lo/-^) expressed Bhlhe41 and were identical to splenic transitional B-1a progenitors (CD19^+^CD93^+^B220^lo/-^CD5^+^), which only gave rise to peritoneal B-1a cells. Moreover, we identified novel neonatal splenic CD19^hi^dTomato^+^B220^hi^CD43^lo^CD5^lo^ B cells, which differentiated to peritoneal B-1a and B-1b cells and were increased upon loss of *Bhlhe41*.

## Materials and methods

### Mice

*Bhlhe41^dTomato-Cre^* mice at C57BL/6 background were generated by Cyagen Biosciences Inc using CRISPR-Cas9 technology. Briefly, a targeting vector with a *dTomato-2A-Cre-polyA* cassette inserted upstream of the *Atg* start site of *Bhlhe41* locus was constructed with homology arms generated using BAC clone RP24-71C23 and RP23-393E18 from the C57BL/6 library as template. A guide RNA (gRNA) 5’ *CAGCCATTGAACATGGACGAAGG* 3’ was co-injected with Cas9 and the targeting vector into fertilized eggs for mice production. The founder pups were genotyped by PCR followed by DNA sequencing of PCR products. A common forward primer 5’ *ATACTGCACTGAAGAGGGAGAGC* 3’ combined with a reverse primer for wild-type allele 5’ *TCGCTTCAAGCTCCTTTTGG* 3’ (PCR product size 366 bp) or for transgenic allele 5’ *CTTGGAGCCGTACATGAACTG* 3’ (PCR product size 339 bp) were used for genotyping. The *Bhlhe41^dTomato-Cre^* mice can be shared for research purposes on request.

*Rag2^-/-^* mice (Stock ID: 008449), *B6 Cd45.1* congenic mice (*B6.SJL*, Stock ID: 002014)*, Rosa26^EYFP^* (Stock ID: 006148), and *Rosa26^iDTR^* mice (Stock ID: 007900) were obtained from the Jackson Laboratory. All mice were on the C57BL/6 genetic background and maintained in a specific pathogen-free (SPF) facility of Xuzhou Medical University. All animal studies were performed in accordance with the protocol approved by the Animal Experimental Ethics Committee of Xuzhou Medical University.

### Reagents

Anti-mouse CD3ϵ-FITC (145-2C11), anti-mouse CD4-Brilliant Violet 510 or FITC (GK1.5), anti-mouse CD8-APC or FITC (53-6.72), anti-mouse CD11b-APC (M1/70), anti-mouse CD11c-Pacific Blue (N418), anti-mouse NK1.1-PE-Cy7 (PK136), anti-mouse CD19-Pacific Blue or APC (1D3), anti-mouse CD5-FITC or APC (53-7.3), anti-mouse CD43-APC or PE-Cy7 (S11), anti-mouse B220- Brilliant Violet 510 (RA3-6B2), anti-mouse CD45-Percp-Cy5.5 (I3/2.3), anti-mouse CD45.2-Percp Cy5.5 (104.2), anti-mouse Ly-6G-Pacific Blue (1A8), anti-mouse IgD-Alexa fluor 647 (11-26c), anti-mouse CD24-PE-Cy7(M1/69), anti-mouse MHC-II-APC (M5/114.15.2), anti-mouse F4/80-Percp-Cy5.5 (QA17A29), anti-mouse TER119-Pacific Blue (TER-119), anti-mouse SiglecF-APC (S17007L), anti-mouse CX3CR1-APC (QA16A03), anti-mouse TCRβ-Pacific Blue (H57-597), purified anti-mouse CD40 (1C10), purified anti-mouse IFN-γ (XMG1.2), purified anti-mouse IL-4 (11B11), anti-mouse Sca-1-FITC (E13-161.7), anti-mouse cKit (ACK2)-PE-Cy7, and Zombie Aqua fixable viability kit (CAT: 423102) were purchased from Biolegend. Anti-mouse BST2-APC (eBio927), anti-mouse IgM-eFluor 450, anti-mouse IL-4-APC (11B11), anti-mouse IL-17A-APC (TC11-18H10.1), anti-mouse IFN-γ-PE (XMG1.2), anti-mouse IL-13-PE (eBio13A), ionomycin (CAT: 24222), 1000 × Monensin (CAT: 00-4505-51), TMB solution (CAT: 00-4201-56), ProLong Diamond Antifade Mountant with DAPI (CAT: P36962), goat anti-rabbit Alexa Flour 488 (CAT: A-11034), goat anti-rat Alexa Flour 594 (CAT: A11007), donkey anti-rabbit Alexa Flour 555(CAT: A31572), and rabbit anti-goat Alexa Flour 488 (CAT: A27012) were purchased from Thermo Fisher Scientific. NP-BSA (CAT: N-5050H-100) and NP-Ficoll (CAT: F-1420-100) were purchased from Biosearch Technologies. Goat anti-mouse IgM-HRP (CAT: 1020-05), goat anti-mouse IgA-HRP (CAT: 1040-05), goat anti-mouse IgG3-HRP (CAT: 1100-05), and goat anti-mouse IgG-HRP (CAT: 1030-05) were purchased from Southern Biotech. AffiniPure F(ab’)2 Fragment Goat anti-mouse IgM (CAT: 115-006-075) was purchased from Jackson ImmunoResearch. Recombinant murine IL-4 (CAT: 214-14), recombinant murine IL-12 (CAT: 210-12), recombinant murine IL-2 (CAT: 212-12), recombinant human TGF-β (CAT: 100-21), and recombinant murine IL-6 (CAT: 216-16) were purchased from PeproTech. Phorbol 12-myristate 13-acetate (PMA, CAT: 1201/1) was purchased from TOCRIS. Diphtheria toxin from *Corynebacterium diphtheriae* (CAT: 150) was purchased from ListBio. Rabbit anti-Iba-1 (CAT: 019-19741) was purchased from Wako. Rat anti-CD68 (CAT: ab53444) was purchased from Abcam. Rabbit anti-RFP (CAT: 600-401-379) was purchased from Rockland. Goat anti-tdTomato (CAT: orb182397) was purchased from Biorbyt.

### Flow cytometric analysis

For characterization of dTomato and EYFP in adult *Bhlhe41^dTomato-Cre^* and *Bhlhe41^dTomato-Cre/^* ^+^ *Rosa26^EYFP^* mice, various immune cells across multiple sites including thymus, spleen, bone marrow, brain, liver, bronchoalveolar lavage fluid (BALF), and peritoneal cavity lavage fluid were identified by flow cytometric analysis with markers as follows: pro B cells (B220^+^CD43^+^IgM^–^IgD^–^), pre B cells (B220^+^CD19^+^CD43^-^IgM^-^IgD^-^), immature B cells (B220^+^CD19^+^CD43^-^IgM^+^IgD^-^), circulating B cells (B220^+^CD19^+^CD43^-^IgM^+^IgD^+^), neutrophils (CD11b^+^Ly-6G^+^ Ly-6C^-^), and monocytes (Lin^-^CD11b^+^Ly-6G^-^Ly-6C^+^) in bone marrow, double-negative (DN) thymocytes (CD45^+^CD4^-^CD8^-^), double-positive (DP) thymocytes (CD45^+^CD4^+^CD8^+^), CD4 single-positive (CD4 SP) thymocyte (CD45^+^CD4^+^CD8^-^), and CD8 single-positive (CD8 SP) thymocyte (CD45^+^CD4^-^CD8^+^) in thymus, splenic CD4^+^ T cells (CD45^+^CD4^+^CD8^-^), splenic CD8^+^ T cells (CD45^+^CD8^+^CD4^-^), splenic follicular B cells (FoB, B220^+^IgD^+^IgM^-/lo^), splenic marginal zone B cells (MZB, B220^+^IgD^-/lo^IgM^+^), splenic B-1 cells (CD3ϵ^-^TER119^-^NK1.1^-^Ly6G^-^CD11c^-^CD19^+^B220^lo/-^), alveolar macrophage (CD45^+^CD11c^+^SiglecF^+^), microglia (CD45^lo^CD11b^+^), peritoneal B-1a (CD19^+^B220^lo/-^CD43^+^CD23^-^CD5^+^), peritoneal B-1b (CD19^+^B220^lo/-^CD43^+^CD23^-^CD5^-^), peritoneal B-2 cells (CD19^+^B220^+^CD43^-^CD23^+^), and neonatal splenic transitional B-1a cells (CD19^+^CD93^+^B220^lo/-^CD43^+^CD5^+^).

### Immunofluorescence analysis

Thymus, lung, spleen, liver, kidney, small intestine, and brain from *Bhlhe41^dTomato-Cre^* mice were collected after transcardial perfusion with PBS. Tissues were fixed overnight in 4% PFA followed by dehydration in 30% sucrose. Dehydrated tissues were embedded in Tissue-Tek OCT and 9 μm cryosections were obtained for immunofluorescence analysis. Following being blocked with PBS containing 5% bovine serum albumin and being permeabilized with 0.3% Triton-X 100 in blocking buffer, sections were incubated with primary antibodies overnight at 4°C as follows: rabbit anti-RFP (1:200), rat anti-CD68 (1:800), rabbit anti-Iba-1 (1:500), and goat anti-tdTomato (1:200). After washing, sections were incubated with secondary antibodies for 2 h at room temperature as follows: goat anti-rabbit Alexa Flour 488 (1:500), goat anti-rat Alexa Flour 594 (1:500), donkey anti-rabbit Alexa Flour 555 (1:500), and rabbit anti-goat Alexa Flour 488 (1:500). Coverslips were mounted with ProLong Diamond Antifade Mountant with DAPI. Images were taken using a conventional fluorescence microscope.

### *In vitro* T- and B-cell differentiation

EYFP^-^ naïve splenic B cells (B220^+^CD43^-^) were isolated from *Bhlhe41^dTomato-Cre/+^Rosa26^EYFP^* mice and wild-type mice and cultured *in vitro* with LPS (1 μg/ml), with anti-IgM (10 μg/ml) + IL-4 (10 ng/ml) and anti-CD40 (10 μg/ml) + IL-4 (10 ng/mL) for 3 days. *In vitro* mouse Th1, Th2, and Th17 cell differentiation was performed with EYFP^-^ naïve CD4^+^ T cells (CD4^+^CD25^-^CD62L^+^CD44^-^) purified from wild-type and *Bhlhe41^dTomato-Cre/+^Rosa26^EYFP^* mice as previously described (13). dTomato and EYFP were analyzed directly by flow cytometric analysis of *in vitro* differentiated Th1 and Th17 cells at day 3 and Th2 cells at day 5 post-differentiation. PMA (100 μg/ml), ionomycin (0.5 μg/ml), and 1× Monensin were added to *in vitro* differentiated T cells 5 h prior to flow cytometric analysis of intracellular IFN-γ and IL-17 for Th1- and Th17 cells, as well as IL-4 and IL-13 for Th2 cells. EYFP and dTomato expressions were directly analyzed by flow cytometric analysis after exclusion of dead cells stained with Zombie Aqua dye.

### Depletion of B-1 cells and immunization

For depletion of B-1 cells, *Bhlhe41^dTomato-Cre/+^Rosa26^iDTR^* mice were obtained by crossing *Bhlhe41^dTomato-Cre^* mice with *Rosa26^iDTR^* mice. *Bhlhe41^dTomato-Cre/+^Rosa26^iDTR^* and *Bhlhe41^dTomato-Cre/+^* mice were injected with a single dose of diphtheria toxin (DT, 2 ng/g or 4 ng/g), and the depletion efficacy was confirmed by flow cytometric analysis of dTomato^+^ cells at days 2 and 4 post-DT injection. For immunization, *Bhlhe41^dTomato-Cre/+^Rosa26^iDTR^* and *Bhlhe41^dTomato-Cre/+^* mice were injected with two doses of DT (2 ng/g) at days 0 and 4 and immunized with 50 μg of NP-Ficoll in 200 μl of PBS at day 1. Mice were sacrificed at day 8 for ELISA measurement of NP-specific IgM, IgA, IgG, and IgG3.

### ELISA

ELISA measurement of NP-specific IgM, IgA, IgG, and IgG3 was performed by coating ELISA plates with 500 ng of NP-BSA per well overnight at 4°C. Following washing with PBS containing 0.05% Tween 20 and blocking with 1×ELISA Diluent for 1 h, diluted serum was added and incubated for 2 h at room temperature before washing with PBS containing 0.05% Tween 20. HRP-coupled IgM, IgA, IgG, and IgG3 were added and incubated for 1 h followed by washing and addition of TMB solution sequentially. A 2 N sulfuric acid solution was used to stop the reaction prior to plate reading at OD450.

### Adoptive cell transfer

For the characterization of B-1a cell development in *Bhlhe41*-deficient mice, bone marrow cells from adult *B6.SJL* and *Bhlhe41^dTomato-Cre/dTomato-Cre^* mice were mixed equally and adoptively transferred to irradiated *Rag2^-/-^* mice (6 Gy) to generate *B6.SJL* and *Bhlhe41^dTomato-Cre/dTomato-Cre^* chimeric mice. Chimeric mice were sacrificed after 6 weeks for flow cytometric analysis of peritoneal B-1 cells derived from donor *B6.SJL* and *Bhlhe41^dTomato-Cre/dTomato-Cre^* mice. For the characterization of neonatal dTomato^+^ B cells, splenocytes from 1-week-old *Bhlhe41^dTomato-Cre/+^* mice were sorted as CD19^+^dTomato^+^CD43^hi^B220^lo^ and CD19^+^dTomato^+^CD43^lo^B220^hi^ subsets and subsequently *i.p*. transferred into 3-week-old *B6.SJL* congenic mice (20000 cells/recipient mouse). The recipient mice were sacrificed in 5 days post-transfer. Peritoneal B-1a (CD19^+^B220^lo/-^CD43^+^CD5^+^), B-1b (CD19^+^B220^lo/-^ CD43^+^CD5^-^), and B-2 cells (CD19^+^ B220^+^CD43^-^) were examined by flow cytometric analysis.

### Statistical analysis

All statistical analyses were performed using GraphPad Prism 6 (GraphPad). Differences between two groups were analyzed by Student’s t-test. For multiple comparisons, one-way or two-way ANOVA followed by Bonferroni multiple comparison test was used. Data were expressed as mean ± SD. A *P* value < 0.05 was considered significant, and the level of significance was indicated as *P < 0.05.

## Results

### B-1 cell-specific expression of *Bhlhe41* in B-cell lineage

**Figure 1:**
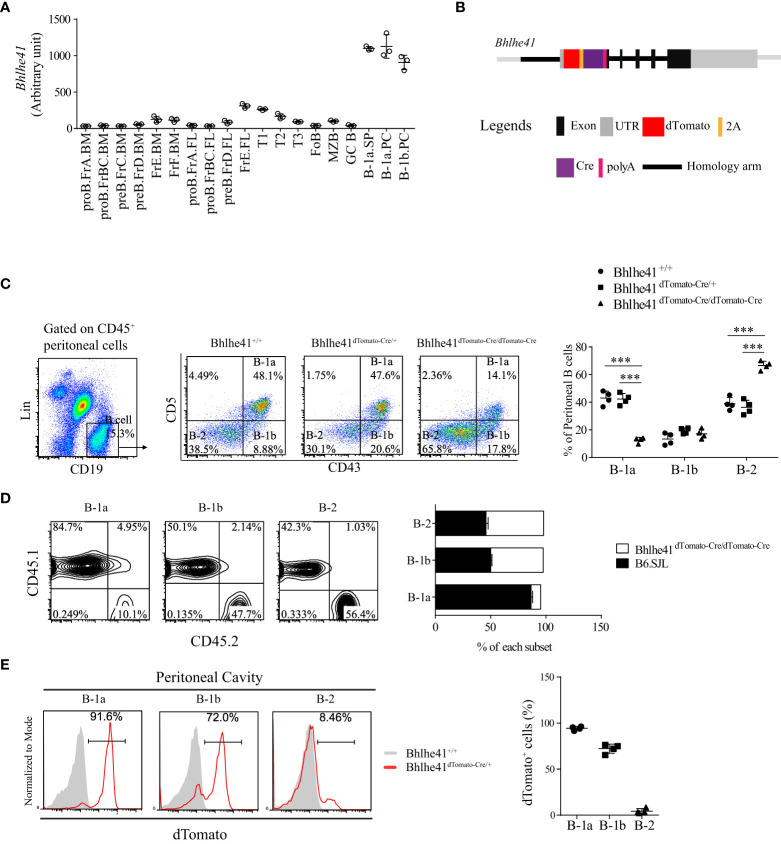
Generation of *Bhlhe41* reporter and knockout mouse model *Bhlhe41^dTomato-Cre^*. **(A)** Analysis of *Bhlhe41* expression in different stages of B cells using a public gene expression microarray data (GSE15907) after quantile normalization. **(B)** Targeting construct for the *Bhlhe41* locus with a *dTomato-2A-Cre-polyA* cassette inserted upstream of the *Atg* start site of *Bhlhe41* locus. **(C)** Flow cytometric analysis of peritoneal B cells from adult *Bhlhe41^+/+^, Bhlhe41^dTomato-Cre/+^*, and *Bhlhe41^dTomato-Cre/dTomato-Cre^* mice. The gating strategy for each type is illustrated on the left panel. One-way ANOVA followed by Bonferroni multiple comparison test was used for the statistical analysis. **(D)** Flow cytometric analysis of dTomato expression in peritoneal B cells from adult *Bhlhe41^+/+^, Bhlhe41^dTomato-Cre/+^*, and *Bhlhe41^dTomato-Cre/dTomato-Cre^* mice. ***P < 0.001. **(E)** Flow cytometric analysis of dTomato in peritoneal B cells from adult *Bhlhe41^+/+^* and *Bhlhe41^dTomato-Cre/+^* mice.

It has been reported that transcription factor Bhlhe41 is highly expressed in B-1 cell, alveolar macrophage, microglia, and Th2 cell and is critical for B-1a cell development and self-renewal (10, 14, 15). To fully understand the specificity of Bhlhe41 in immune system, we analyzed *Bhlhe41* expression in murine multiple immune lineages using a public gene expression microarray data of long-term (LT) and short-term (ST) hematopoietic stem cells (HSC), multi-lymphoid progenitor (MLP), monocyte-dendritic cell progenitor (MDP), granulocyte-macrophage progenitor (GMP), megakaryocyte-erythrocyte progenitor (MEP), common lymphoid progenitor (CLP), common dendritic cell progenitor (CDP), T cell, B cell, monocyte, macrophage, dendritic cell, neutrophil, microglia, natural killer cell, and natural killer T cell by the Immunological Genome Project (16). The *Bhlhe41* transcript was not detected in hematopoietic progenitors, as well as peripheral T cells, monocyte, dendritic cell, neutrophil, natural killer cell, and natural killer T cell (**Figure S1**). In agreement with previous reports, splenic and peritoneal B-1a and B-1b cell, alveolar macrophage, and microglia highly expressed *Bhlhe41* (**Figure 1A**, **Figure S1**). In B-cell lineage, *Bhlhe41* was expressed to a much lesser extent in immature and circulating B cells (fractions E and F) from bone marrow, transitional 1 (T1) and T2 B cells, and marginal zone B cells (MZB), compared to splenic and peritoneal B-1a and B-1b cells (**Figure 1A**). Hence, *Bhlhe41* is preferentially and highly expressed in B-1 cells in B-cell lineage, and *Bhlhe41* locus may be suitable for generating a transgenic mouse model for fate mapping and functional analysis of B-1 cells.

### Generation of *Bhlhe41*-reporter and knockout mouse model *Bhlhe41^dTomato-Cre^*

We generated a bacterial artificial chromosome (BAC) transgenic *Bhlhe41*-reporter mouse by insertion of a *dTomato-2A-Cre-polyA* cassette upstream of the *Atg* start site of *Bhlhe41* locus using CRISPR-Cas9 technology (**Figure 1B**). The *2A*-encoded sequence mediates a co-translation “cleavage” of polyproteins in eukaryotic cells (17). Hence, the introduction of *2A* between *dTomato* and *Cre* resulted in independent co-expression of dTomato and Cre recombinase driven by the *Bhlhe41* promoter, respectively (**Figure 1B**). As a result, dTomato can be used to trace Bhlhe41 expression, while *Bhlhe41* promoter-driven Cre recombinase will enable the deletion of the gene of interest in cells that express or once expressed *Bhlhe41*. In addition, the insertion of *polyA* upstream of the *Atg* start site of the *Bhlhe41* locus led to early termination of the Bhlhe41 transcription and thereby made *Bhlhe41^dTomato-Cre/dTomato-Cre^* mice be *Bhlhe41* knockout mice (**Figure 1B**).

The correct insertion of the *dTomato-2A-Cre-polyA* cassette upstream of the *Atg* start site of the *Bhlhe41* locus was confirmed by DNA sequencing (**Figure S2**). *Bhlhe41^dTomato-Cre^* mouse was further phenotypically validated by flow cytometric analysis of peritoneal B cells, which showed a significant decreased percentage of peritoneal B-1a cells in *Bhlhe41^dTomato-Cre/dTomato-Cre^* mice (**Figure 1C**). Adoptive transfer of an equal number of bone marrow cells from *B6.SJL* (CD45.1^+^) and *Bhlhe41^dTomato-Cre/dTomato-Cre^* mice (CD45.2^+^) to irradiated *Rag2^-/-^* mice also confirmed the defect in the generation of peritoneal B-1a cells rather than B-1b and B-2 cells (**Figure 1D**). These data are in agreement with the previous report of an essential role of Bhlhe41 in regulating B-1a cell self-renewal (10). In addition, we validated *Bhlhe41^dTomato-Cre^* mice by flow cytometric analysis of dTomato in peritoneal B cells, which were highly detected in peritoneal B-1a and B-1b cells rather than B-2 cells (**Figure 1E**). These data collectively indicate that we have successfully generated *Bhlhe41-*reporter and knockout mouse.

### Identification of neonatal B-1 progenitors as transitional B-1a progenitors that highly express Bhlhe41 using *Bhlhe41^dTomato-Cre^* mice

To comprehensively gain the knowledge of Bhlhe41 expression, we examined Bhlhe41 expression in multiple immune and non-immune organs by immunofluorescence analysis of dTomato in spleen, thymus, brain, lung, liver, kidney, and small intestine from *Bhlhe41^dTomato-Cre^* mice (**Figure S3**). dTomato was detected to a very limited extent in spleen and small intestine but was barely detectable in thymus, liver, and kidney (**Figures S3A**). In contrast, co-expression of dTomato with alveolar macrophage marker CD68 in lung and with microglia marker Iba-1 in brain was observed (**Figures S3B, C**). This is in line with the gene expression microarray data of preferential *Bhlhe41* expression in alveolar macrophage and microglia (**Figure 1A**). Therefore, Bhlhe41 is preferentially expressed in B-1 cell, alveolar macrophage, and microglia.

**Figure 2:**
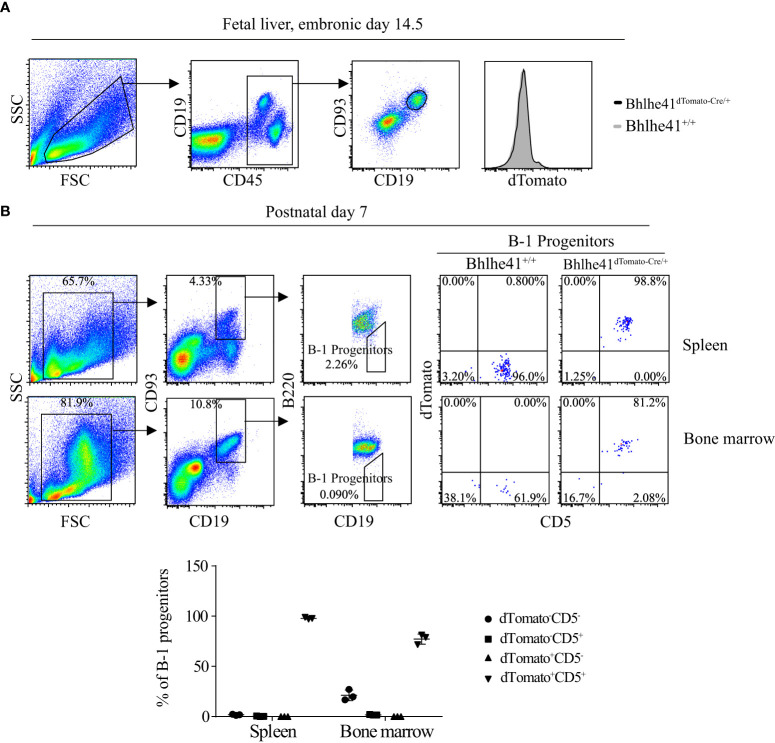
Identification of neonatal B-1 progenitors as transitional B-1a progenitors that highly express Bhlhe41 using *Bhlhe41^dTomato-Cre^* mice. Flow cytometric analysis of dTomato expression in **(A)** B cells in fetal liver from *Bhlhe41^+/+^* and *Bhlhe41^dTomato-Cre/+^* mice at embryonic day 14.5 and **(B)** B-1 progenitors (CD19^+^CD93^+^B220^lo/-^) in spleen and bone marrow from *Bhlhe41^+/+^* and *Bhlhe41^dTomato-Cre/+^* mice at postnatal day 7. The gating strategy for each type is illustrated on the left panel.

B-1 cell development occurs in the fetal liver, whereas B-1 progenitors from the bone marrow and spleen of neonatal mice also contribute to adult B-1 cell pool (1). To further understand the earliest stage of B-1 cell development that can be monitored and targeted by *Bhlhe41^dTomato-Cre^* mouse, we analyzed Bhlhe41 expression in B cells in the fetal liver from *Bhlhe41^dTomato-Cre^* mice at embryonic day 14.5. In agreement with the previous study, dTomato was not detectable in fetal B cells (Lin^-^CD93^+^CD19^+^) (**Figure 2A**) (10). We next examined B-1 progenitors (CD19^+^CD93^+^B220^lo/-^) in the spleen and bone marrow from neonatal mice at postnatal day 7, which showed that more than 95% of splenic B-1 progenitors and more than 80% of B-1 progenitors in bone marrow were dTomato^+^CD5^+^ (**Figure 2B**). Moreover, dTomato was completely associated with CD5 on B-1 progenitors (**Figure 2B**). These data indicate that neonatal B-1 progenitors (CD19^+^CD93^+^B220^lo/-^) are identical to well-defined transitional B-1a progenitors (CD19^+^CD93^+^B220^lo/-^CD5^+^), which highly express Bhlhe41.

Previously, neonatal splenic B-1 progenitors are also identified as CD19^hi^cKit^+^Sca1^+^ B cells, which can be induced through stimulation of adult splenic B-1 progenitors (CD19^hi^B220^lo/-^) by LPS (8). We, therefore, also investigated the association of Bhlhe41, CD19, Sca1, and cKit on splenic B cells from *Bhlhe41^+/+^* and *Bhlhe41^dTomato-Cre^^/+^* mice at postnatal day 7. Sca-1 and dTomato were strictly expressed in CD19^hi^ B cells, and the majority of dTomato^+^ B cells were Sca-1^+^ (**Figure S4**). As expected, CD19^hi^cKit^+^Sca1^+^ B-1 progenitors are dTomato^+^ (**Figure S4**), indicating that neonatal B-1 progenitors, identified as CD19^hi^cKit^+^Sca1^+^ cells, express Bhlhe41.

### *Bhlhe41^dTomato-Cre^* mouse can be used to efficiently target B-1 cells *in vivo*

**Figure 3:**
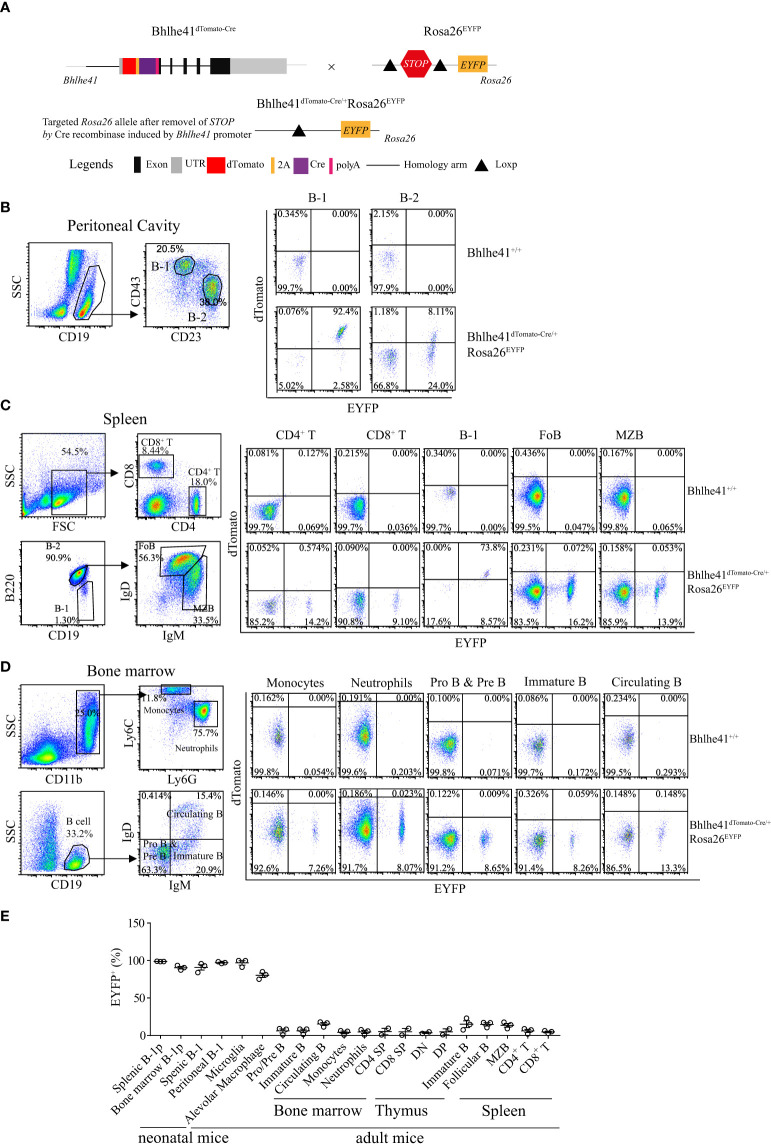
*Bhlhe41^dTomato-Cre^* mouse can be used to efficiently target B-1 cells *in vivo*. **(A)** Generation of *Bhlhe41^dTomato-Cre/^* ^+^ *Rosa26^EYFP^* mice for analyzing the efficacy and specificity of deletion of *Loxp*-flanked *STOP* sequence driven by Cre recombinase induced by *Bhlhe41* promoter. Flow cytometric analysis of dTomato and EYFP in **(B)** peritoneal B-1 and B-2 cells, **(C)** splenic CD4^+^ T cells, CD8^+^ T cells, B-1 cells, follicular B (FoB) cells, and marginal zone B (MZB) cells, **(D)** neutrophils, monocytes, Pro and Pre B cells, immature B cells, and circulating B cells in the bone marrow from adult *Bhlhe41^dTomato-Cre/^* ^+^ *Rosa26^EYFP^* mice. The gating strategy for each type is illustrated on the left panel. **(E)** The summary of EYFP^+^ cells in B-1 progenitors (B-1p) from neonatal *Bhlhe41^dTomato-Cre/^* ^+^ *Rosa26^EYFP^* mice at postnatal day 7 and in different immune cells from adult *Bhlhe41^dTomato-Cre/^* ^+^ *Rosa26^EYFP^* mice. CD4 SP, CD4 single positive; CD8 SP, CD8 single positive; DN, double negative; DP, double positive.

Next, we sought to examine if *Bhlhe41^dTomato-Cre^* mouse can be used for Cre recombinase-mediated efficient deletion of *Loxp* sites in B-1 cells. To test this, we generated *Bhlhe41^dTomato-Cre/^* ^+^ *Rosa26^EYFP^* mice by crossing *Bhlhe41^dTomato-Cre/^* ^+^ mice with *Rosa26^EYFP^* mice, the latter of which have a *Loxp*-flanked *STOP* sequence followed by *EYFP* downstream of the *Gt(ROSA)26Sor* locus (**Figure 3A**). As EYFP is irreversibly and persistently expressed upon the removal of *Loxp*-flanked *STOP* sequence by Cre recombinase, cells that express or transiently expressed Bhlhe41 and Cre recombinase in *Bhlhe41^dTomato-Cre/^* ^+^ *Rosa26^EYFP^* mice can be identified as dTomato^+^EYFP^+^ and dTomato^-^EYFP^+^ cells, respectively.

We firstly performed flow cytometric analysis of dTomato and EYFP in immune cells in the thymus, bone marrow, spleen, and peritoneal cavity from adult *Bhlhe41^dTomato-Cre/^* ^+^ *Rosa26^EYFP^* mice (**Figures 3B-E**, **Figure S5**). Consistent with the previous report (10), more than 90% of peritoneal B-1 cells (CD19^+^CD43^+^CD23^-^) and less than 10% of peritoneal B-2 cells (CD19^+^CD43^-^CD23^+^) express Bhlhe41, evidenced by co-expression of dTomato and EYFP (**Figure 3B**). Of note, approximately 25% of peritoneal B-2 cells and 2% of peritoneal B-1 cells were dTomato^-^EYFP^+^, indicating that these cells may transiently express Bhlhe41 and Cre recombinase (**Figure 3B**). Similar to peritoneal B-1 cells, approximately 75% and 10% of splenic B-1 cells (Lin^-^CD19^+^B220^lo/-^) express and transiently expressed Cre recombinase, respectively (**Figure 3C**). As expected, Cre recombinase was highly expressed in alveolar macrophage and microglia, evidenced by more than 75% of EYFP^+^ alveolar macrophage and 99% of EYFP^+^ microglia in *Bhlhe41^dTomato-Cre/^* ^+^ *Rosa26^EYFP^* mice (**Figures S5A, B** and **Figure 3E**).

In line with the microarray data (**Figure 1A**), less than 1% of dTomato^+^EYFP^+^ cells were detected in thymocytes including double-negative (DN, CD4^-^CD8^-^), double-positive (DP, CD4^+^CD8^+^), CD4 single-positive (CD4 SP), and CD8 single-positive (CD8 SP) thymocytes (**Figure S5C** and **Figure 3E**), splenic CD4^+^ T cells and CD8^+^ T cells, monocytes, and neutrophils (**Figures 3C, D**), indicating that Bhlhe41 is not expressed in these cells. However, approximately 15% or less of these cells were dTomato^-^EYFP^+^, indicating that Bhlhe41 and Cre recombinase may be once transiently expressed in these cells (**Figure S5C**, **Figures 3C–E**).

Although *Bhlhe41* is detected in immature and circulating B cells (fractions E and F) from bone marrow and marginal zone B cells (**Figure 1A**), less than 1% of dTomato^+^EYFP^+^ cells were detected in Pro- and Pre-B (CD19^+^IgD^-^IgM^-^), immature B (CD19^+^IgD^-^IgM^+^), and circulating B cells (CD19^+^IgD^+^IgM^+^) from bone marrow, splenic follicular B cells (CD19^+^IgD^+^IgM^lo/-^), and marginal zone B cells (CD19^+^IgD^lo/-^IgM^+^) (**Figures 3C–E**), indicating that Bhlhe41 is not expressed by these B cells. However, similar to other immune cells, approximately 15% dTomato^-^EYFP^+^ cells or less were observed in these B subsets, indicating that these cells may once transiently express Bhlhe41 and Cre recombinase (**Figures 3C–E**).

Bhlhe41 is expressed in neonatal B-1a progenitors (**Figure 2B**) (10). As expected, flow cytometric analysis of EYFP in *Bhlhe41^dTomato-Cre/^* ^+^ *Rosa26^EYFP^* mice at postnatal day 7 showed Cre-mediated recombination in more than 95% of neonatal B-1 progenitors (CD19^+^CD93^+^B220^lo/-^) in bone marrow and spleen (**Figure 3E**). These data collectively indicate that *Bhlhe41^dTomato-Cre^* mouse can be used for targeting B-1 cells *in vivo* as early as from the stage of neonatal B-1 progenitors.

### *In vitro*–activated follicular B cells and differentiated Th2 cells are not targeted by *Bhlhe41^dTomato-Cre^* mouse for lacking Bhlhe41

**Figure 4:**
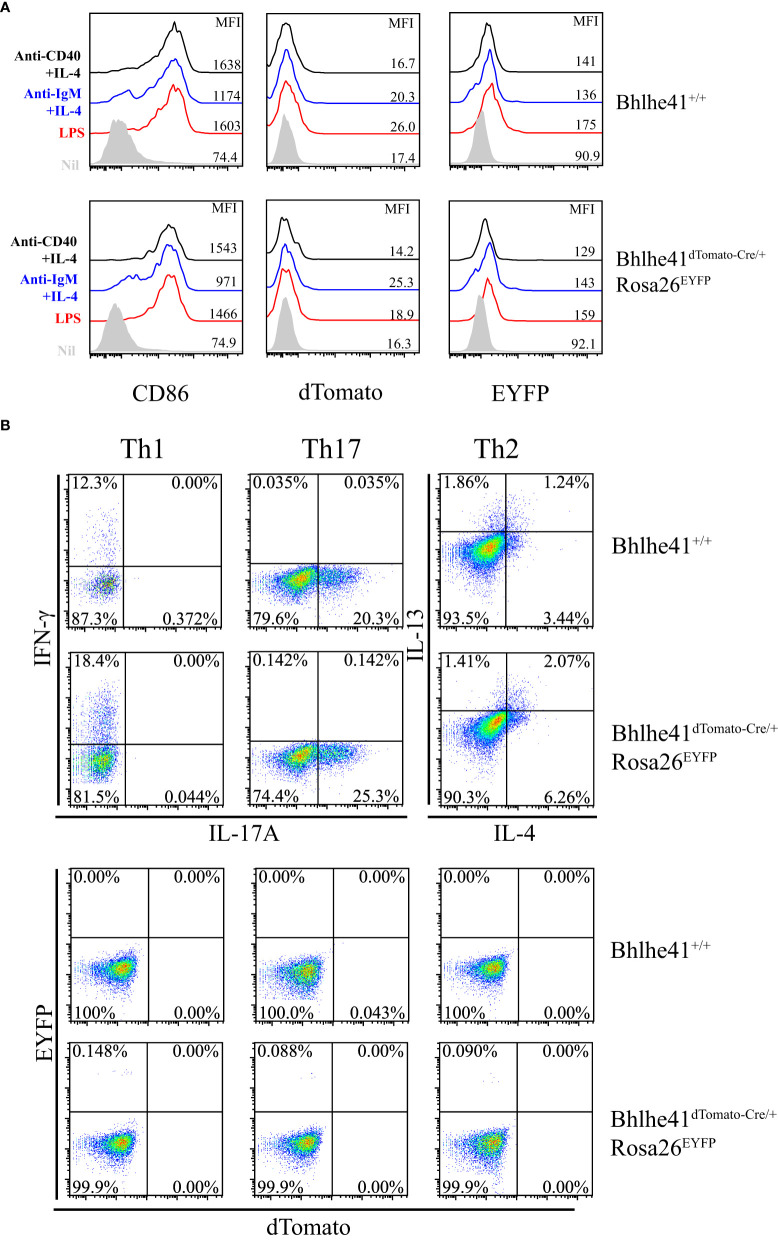
*In vitro–*activated follicular B cells and differentiated Th2 cells do not express Bhlhe41 and are not targeted by *Bhlhe41^dTomato-Cre^* mouse. **(A)** EYFP^-^ follicular B cells (B220^+^CD43^-^) were purified from *Bhlhe41^+/^* ^+^ and *Bhlhe41^dTomato-Cre/^* ^+^ *Rosa26^EYFP^* mice and *in vitro* cultured with LPS, anti-IgM plus IL-4, and anti-CD40 plus IL-4, respectively. EYFP, dTomato, and CD86 were assayed by flow cytometric analysis. **(B)** EYFP^-^ naïve CD4^+^ T cells (CD4^+^CD25^-^CD62L^+^CD44^-^) were purified from *Bhlhe41^+/^* ^+^ and *Bhlhe41^dTomato-Cre/^* ^+^ *Rosa26^EYFP^* mice and *in vitro* polarized toward Th1, Th2, and Th17 subsets. EYFP and dTomato were directly examined by flow cytometric analysis.

Bhlhe41 is induced in follicular B cells upon activation by multiple modulators including LPS, anti-IgM plus IL-4, and anti-CD40 plus IL-4 (10). Therefore, we asked if this may limit the utilization of *Bhlhe41^dTomato-Cre^* mouse for specifically targeting B-1 cell rather than B-2 cell. To address this, EYFP^-^ follicular B cells (B220^+^CD43^-^) were purified from *Bhlhe41^dTomato-Cre/^* ^+^ *Rosa26^EYFP^* and *in vitro*–cultured with LPS, anti-IgM plus IL-4, and anti-CD40 plus IL-4, respectively. Although B-cell activation marker CD86 significantly increased on activated follicular B cells, no induction of dTomato was observed (**Figure 4A**), indicating that *in vitro* activated follicular B cells do not express Bhlhe41. Moreover, EYFP was also not induced in activated follicular B cells from *Bhlhe41^dTomato-Cre/^* ^+^ *Rosa26^EYFP^* mouse, indicating even no transient expression of Bhlhe41 and Cre recombinase in activated follicular B cells (**Figure 4A**).

As Bhlhe41 is induced in *in vitro* differentiated Th2 cells (15), we also examined Cre-mediated recombination of *Loxp* sites in *in vitro* differentiated CD4^+^ T cells from *Bhlhe41^dTomato-Cre/^* ^+^ *Rosa26^EYFP^* mice. To test this, EYFP^-^ naïve CD4^+^ T cells (CD4^+^CD25^-^CD62L^+^CD44^-^) purified from *Bhlhe41^dTomato-Cre/^* ^+^ *Rosa26^EYFP^* were *in vitro* polarized toward Th1, Th2, and Th17 subsets (13). The expression of T-cell subset signature cytokines, namely IFN-γ for Th1 cells, IL-17 for Th17 cells, as well as IL-4 and IL-13 for Th2 cells, were confirmed (**Figure 4B**). However, neither dTomato^+^ nor EYFP^+^ cells were detected in *in vitro* differentiated T cells (**Figure 4B**), indicating that Bhlhe41 and Cre recombinase are not expressed or transiently expressed in *in vitro* polarized Th2 cells.

These data collectively demonstrate that *in vitro*–activated follicular B cells and differentiated Th2 cells are not targeted by *Bhlhe41^dTomato-Cre^* mouse for lacking Bhlhe41.

### Efficient depletion of B-1 cells using *Bhlhe41^dTomato-Cre/+^Rosa26^iDTR^* mice

**Figure 5:**
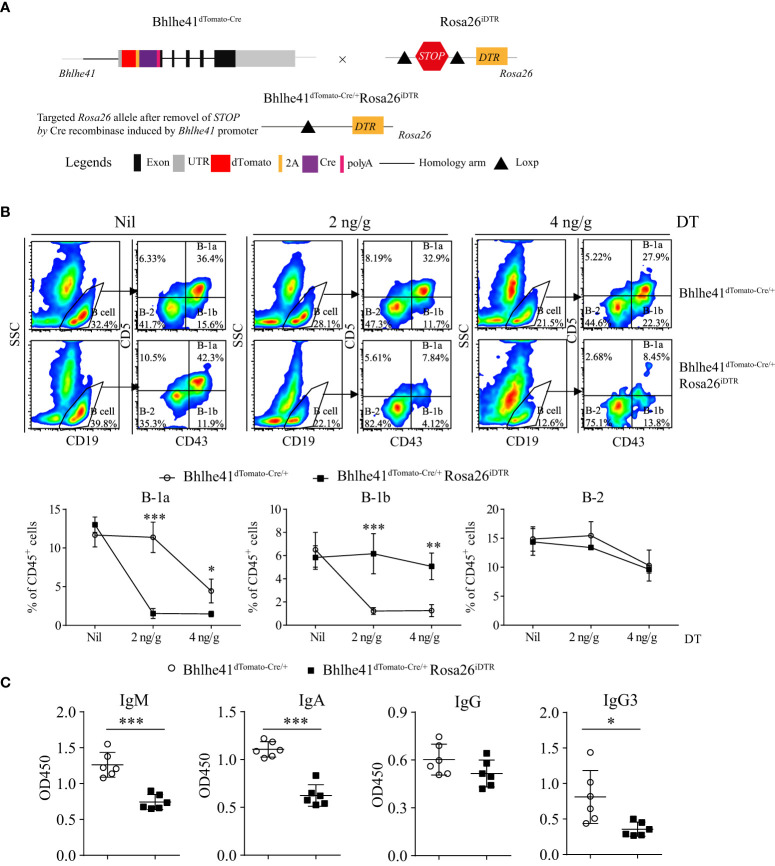
Efficient depletion of B-1 cells using *Bhlhe41^dTomato-Cre/+^Rosa26^iDTR^* mice. **(A)** Generation of *Bhlhe41^dTomato-Cre/^* ^+^ *Rosa26^iDTR^* mice for deletion of B-1 cells. **(B)** Adult *Bhlhe41^dTomato-Cre/^* ^+^ *Rosa26^iDTR^* mice were injected with a single dose of diphtheria toxin (DT) (2 and 4 ng/g) and sacrificed 48 h after DT injection. Immune cells were analyzed by flow cytometric analysis. Two-way ANOVA, followed by Bonferroni multiple comparison test, was used for the statistical analysis. **(C)** Adult *Bhlhe41^dTomato-Cre/^* ^+^ *Rosa26^iDTR^* mice were injected with two doses of DT (2 ng/g) every 3 days and immunized with NP-Ficoll 1 day after the first injection of DT. Serum NP-specific antibodies were analyzed by ELISA. Differences between two groups for each Ig subtype were analyzed by Student’s t-test. *P < 0.05, **P < 0.01, ***P < 0.001.

Previously, depletion of peritoneal B-1 cells is achieved by hypotonic shock through repeated *i.p.* injections of distilled water or by a single injection of mercury chloride for examining the function of B-1 cells in multiple diseases (18–20). To test if *Bhlhe41^dTomato-Cre^* mouse can be utilized for B-1 cell depletion, we generated *Bhlhe41^dTomato-Cre/+^Rosa26^iDTR^* mice by crossing *Bhlhe41^dTomato-Cre^* mouse with *Rosa26^iDTR^* mouse, which has a *Loxp*-flanked *STOP* sequence followed by *DTR* downstream of *Gt(ROSA)26Sor* locus (**Figure 5A**). Upon Cre-mediated removal of *Loxp*-flanked *STOP* sequence, cells that express or transiently expressed Bhlhe41 will express diphtheria toxin receptor and, thereby, can be depleted upon injection of diphtheria toxin (DT). To deplete B-1 cells, we performed a single *i.p.* injection of DT (2 ng/g or 4 ng/g). Flow cytometric analysis of peritoneal B cells revealed that B-1 cells were significantly depleted upon treatment with 2 or 4 ng/g of DT (**Figure 5B**). However, treatment with 4 ng/g of DT also led to decreased B-1 and B-2 cells even in *Bhlhe41^dTomato-Cre^* mice (**Figure 5B**), indicating potential toxic effects on peritoneal B cells. To further confirm B-1 cell depletion, we analyzed NP-specific Ig secretion of DT-treated (2 ng/g) *Bhlhe41^dTomato-Cre^* and *Bhlhe41^dTomato-Cre/+^Rosa26^iDTR^* mice followed by NP-Ficoll immunization, which are known to induce antigen-specific IgM and IgG3. As expected, NP-specific IgM and IgG3 significantly decreased upon DT treatment (**Figure 5C**). In addition, we also noticed a significant reduction of NP-specific IgA (**Figure 5C**). Therefore, *Bhlhe41^dTomato-Cre/+^Rosa26^iDTR^* mice can be used for efficient B-1 cell depletion.

### Identification of CD19^hi^dTomato^+^B220^hi^CD43^lo^CD5^lo^ cells as novel neonatal transitional B-1 progenitors that give rise to B-1a and B-1b cells using *Bhlhe41^dTomato-Cre^* mice

Using *Bhlhe41^dTomato-Cre^* mice, we identified that neonatal splenic B-1 progenitors (CD19^+^CD93^+^B220^lo/-^) expressed Bhlhe41 and were identical to transitional B-1a progenitors (CD19^+^CD93^+^B220^lo/-^CD5^+^) (**Figure 3B**), which are known to only give rise to peritoneal B-1a cells rather than B-1b cells (10). Considering that B-1b progenitors remain elusive and CD19^+^CD93^+^B220^+^ pro B cells from bone marrow can differentiate to both B-2 and B-1 cells in peritoneal cavity (9, 12), we hypothesized that B-1 progenitors may also exist in neonatal CD19^+^CD93^+^B220^+^ B cells that express Bhlhe41.

**Figure 6:**
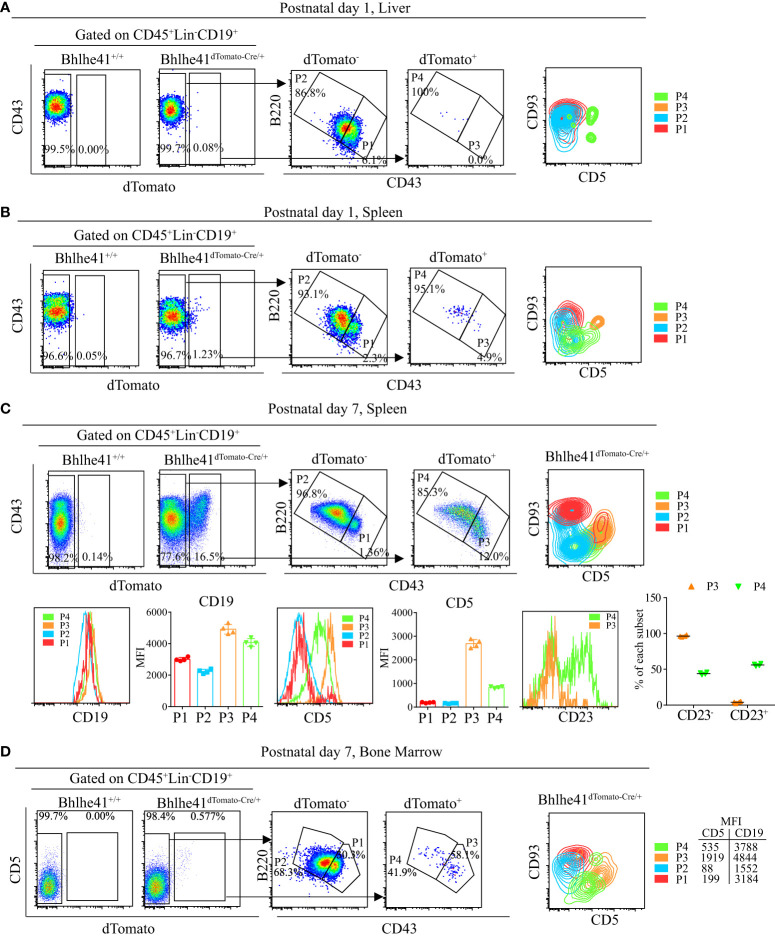
Characterization of neonatal CD19^+^dTomato^+^ B cells using *Bhlhe41^dTomato-Cre^* mice. Flow cytometric analysis of CD19^+^dTomato^+^ B cells in **(A)** liver and **(B)** spleen from *Bhlhe41^dTomato-Cre/+^* mice at postnatal day 1, and **(C)** spleen and **(D)** bone marrow from *Bhlhe41^dTomato-Cre/+^* mice at postnatal day 7. MFI, mean fluorescence intensity.

To test this hypothesis, we analyzed Bhlhe41 expression in total B cells in liver and spleen of neonatal *Bhlhe41^dTomato-Cre^* mice. Approximately 0.1% and 1% of B cells (CD45^+^Lin^-^CD19^+^) from the liver and spleen of *Bhlhe41^dTomato-Cre^* mice at postnatal day 1 were dTomato^+^, respectively (**Figures 6A, B**). Furthermore, characterization revealed that neonatal hepatic dTomato^+^ B cells were mainly B220^hi^CD43^lo^ (P4, **Figure 6A**), whereas neonatal splenic dTomato^+^ B cells were subdivided as B220^lo^CD43^hi^ cells (P3, **Figure 6B**) and B220^hi^CD43^lo^ cells (P4, **Figure 6B**). As splenic B cells from neonatal mice within 1 week after birth mainly are immature transitional B cells (21), we performed further analysis of splenic B cells from *Bhlhe41^dTomato-Cre/+^* mice at postnatal day 7, which showed a significant increase in dTomato^+^ B cells that can also be classified as P3 and P4 (**Figure 6C**). Despite a much lower dTomato^+^ B cells in bone marrow from *Bhlhe41^dTomato-Cre/+^* mice at postnatal day 7, dTomato^+^ B cells can also be subdivided as P3 and P4 cells (**Figure 6D**). The P3 population from both spleen and bone marrow were further identified to be CD93^+^CD5^+^ (**Figures 6B–D**), indicating that P3 are well-defined transitional B-1a progenitors.

B-1 cells express higher CD19 compared to B-2 cells (22). To examine if P4 cells (CD19^+^dTomato^+^B220^hi^CD43^lo^) were also B-1 progenitors, we firstly compared CD19 expression on dTomato^+^ (P3 and P4) and dTomato^-^ (P1 and P2) cells. Similar to P3 cells, P4 cells expressed higher CD19 compared to dTomato^-^ cells (**Figures 6C, D**). Moreover, P4 cells expressed a low level of CD5, which is known to be exclusively expressed on B-1a cells in B-cell lineage (**Figures 6B–D**). As neonatal B cells mainly represent transitional B cells (CD93^+^) that can be subclassified as T1 (CD23^-^) and T2/T3 (CD23^+^) (10, 21), we examined CD23 expression on P3 and P4. In line with the previous report, P3 cells, which were identified as transitional B-1a cells, were mainly at the T1 stage as evidenced by more than 90% of CD23^+^ cells (11). Different from P3 cells, half of P4 cells were CD23^+^, indicating that P4 cells are at the stage of both T1 and T2. In summary, these data indicate that P4 cells (CD19^hi^dTomato^+^B220^hi^CD43^lo^CD5^lo^) may be novel transitional B-1 progenitors that can give rise to peritoneal B-1 cells.

**Figure 7:**
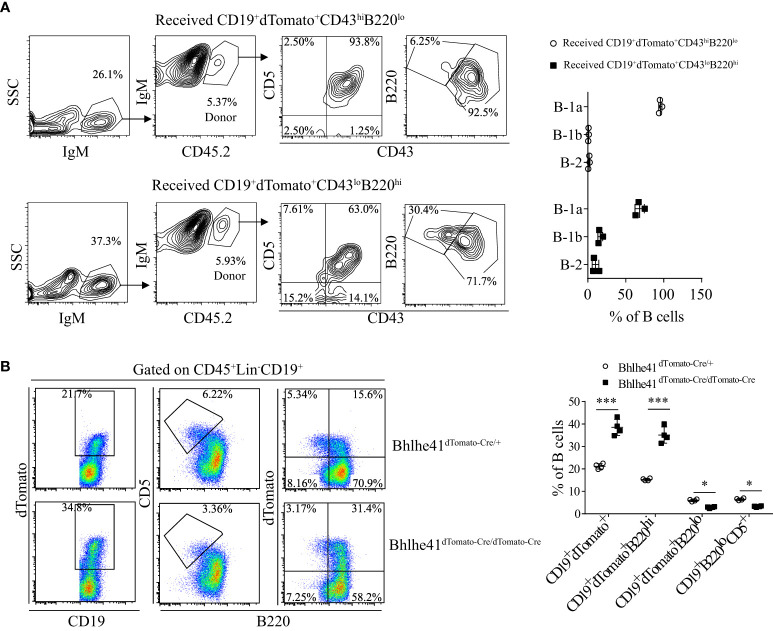
Identification of CD19^+^dTomato^+^B220^hi^CD43^lo^ B cells as novel transitional B-1 progenitors. **(A)** Splenic CD19^+^dTomato^+^B220^hi^CD43^lo^ and CD19^+^dTomato^+^B220^lo^CD43^hi^ B cells were purified from neonatal *Bhlhe41^dTomato-Cre^* mice at postnatal day 7 and *i.p.* adoptively transferred to 3-week *B6.SJL* recipient mice. Flow cytometric analysis of peritoneal B cells in *B6.SJL* recipient mice 5 days after transfer. **(B)** Flow cytometric analysis of splenic B cells from *Bhlhe41^dTomato-Cre^* and *Bhlhe41^dTomato-Cre/dTomato-Cre^* mice at postnatal day 7. Differences between two groups for each cell type were analyzed by Student’s t-test. *P < 0.05, ***P < 0.001.

To further characterize if P4 cells can differentiate to B-1 cells, P3 and P4 cells from *Bhlhe41^dTomato-Cre/+^* mice at postnatal day 7 were sorted with high purity (**Figure S6**) and *i.p.* adoptively transferred to *B6.SJL* recipient mice. Consistent with the previous report, P3 cells, as well-defined transitional B-1a progenitors, exclusively gave rise to peritoneal B-1a cells (**Figure 7A**). Interestingly, P4 cells mainly differentiate to B-1a and B-1b cells with minor contributions to B-2 cells (**Figure 7A**). *Bhlhe41* deficiency impairs neonatal transitional B-1a progenitors (CD19^+^CD93^+^B220^lo/-^CD5^+^) (10). As Bhlhe41 is also expressed in P4 population, we reasoned if Bhlhe41 is involved in regulating P4 populations. To test this, we compared P3 and P4 population in total splenic B cells from *Bhlhe41^dTomato-Cre/+^* and *Bhlhe41^dTomato-Cre/dTomato-Cre^* mice at postnatal day 7. In agreement with the previous study (10), *Bhlhe41* deficiency led to decreased transitional B-1a progenitors (CD19^+^B220^lo/-^CD5^+^ or CD19^+^B220^lo/-^dTomato^+^) (**Figure 7B**). However, we observed a significant increase in CD19^+^dTomato^+^ B cells and CD19^+^B220^hi^dTomato^+^ cells upon loss of *Bhlhe41* (**Figure 7B**). These data collectively indicate that CD19^hi^dTomato^+^B220^hi^CD43^lo^CD5^lo^ B cells are novel neonatal transitional B-1 progenitors that can differentiate to B-1a and B-1b cells.

## Discussion

In the present study, we generated *Bhlhe41^dTomato-Cre^* mouse as a unique Cre transgenic mouse model for targeting the gene of interest in B-1 cells. We showed that an efficient and B-1 cell–specific Cre-recombinase mediated DNA recombination in *Bhlhe41* ^dTomato-Cre/+^ *Rosa26* ^EYFP^ mice, and treatment of *Bhlhe41* ^dTomato-Cre/+^ *Rosa26* ^iDTR^ mice with diphtheria toxin resulted in a robust B-1 cell depletion and a significant decrease in antigen-specific IgM and IgA. Moreover, using *Bhlhe41^dTomato-Cre^* mice, we identified neonatal B-1 progenitors as transitional B-1a progenitors and neonatal splenic CD19^hi^dTomato^+^B220^hi^CD43^lo^CD5^lo^ as novel transitional B-1 progenitors, which differentiated to peritoneal B-1a and B-1b cells.

Although Bhlhe41 is preferentially expressed in B-1 cells and regulates B-1a cell development and self-renewal, how Bhlhe41 is preferentially induced in B-1 cells remains elusive. B-1a cell development is highly dependent on strong B-cell receptor (BCR) signaling, and deficiency of signaling proteins involved in BCR signaling, e.g., Bruton’s tyrosine kinase (BTK) and CD19, is associated with impaired B-1a cell development (10, 23, 24). Kreslavsky et al. reported that the transgenic expression of VH12/Vκ4 BCR, which recognizes phosphatidylcholine presented in host cell membrane and certain pathogens and drives B-1a development, does not rescue the phenotype of impaired B-1a cells upon loss of *Bhlhe41*. Another study by R. Graf et al. recently shows that B-2 cells with BCR changed to VH12/Vκ4 differentiate into *bona fide* B-1 cells that express B-1 cell–specific genes including *Bhlhe41 (* 23). Therefore, it is likely that Bhlhe41 is induced upon the expression of B-1 cell–specific autoreactive BCR and acts downstream of BCR signaling in controlling B-1a cell development and self-renewal.

Adult B-1 cells develop from fetal B-1 progenitors (CD19^+^CD93^+^B220^lo/-^) with contributions from neonatal B-1 progenitors (CD19^+^CD93^+^B220^lo/-^) in spleen and bone marrow (4, 7, 9). In this study, we observed no Bhlhe41 expression in fetal B-1 progenitors. Hence, it is of no doubt that Bhlhe41 is dispensable for the initial generation of fetal B-1 progenitors. Neonatal B-1 progenitors are another important source cells that give rise to adult B-1 cells (10, 11, 21). Neonatal B-1 progenitors (CD19^+^CD93^+^B220^lo/-^) from bone marrow can give rise to both B-1a and B-1b cells, while neonatal splenic transitional B-1a progenitors (CD19^+^CD93^+^B220^lo/-^CD5^+^) exclusively differentiate to B-1a cells (9, 11). In line with these studies, by strictly gating neonatal B-1 progenitors (CD19^+^CD93^+^B220^lo/-^) in *Bhlhe41^dTomato-Cre/^* ^+^ mice, we demonstrated that neonatal splenic B-1 progenitors were identical to transitional B-1a progenitors. However, we observed that neonatal B-1 progenitors from bone marrow include less than 30% of CD5^-^dTomato^-^ cells. As neonatal B-1 progenitors from bone marrow can give rise to both B-1a and B-1b cells, CD5^-^dTomato^-^ neonatal B-1 progenitors may represent a unique population that give rise to B-1 cells. However, it is also possible that CD5^-^dTomato^-^ neonatal B-1 progenitors are non–B-1 progenitors due to cell contamination. Further studies are warranted to elucidate the contribution of these cells to B-1 cell differentiation.

Despite the fact that CD19^+^CD93^+^B220^lo/-^ B cells are identified as B-1 progenitors that give rise to B-1 cells, CD19^+^CD93^+^B220^+^ B cells from bone marrow can also differentiate to peritoneal B-1a and B-1b cells in addition to B-2 cells (9). Moreover, transitional B cells including T1 and T2 B cells (CD19^+^CD93^+^), which predominate in total splenic B cells from neonatal mice within 1 week after birth, preferentially differentiate to B-1a and B-1b cells (21). These studies support the notion of potential B-1 progenitors in neonatal CD19^+^B220^hi^ B cells. Although Bhlhe41 is known to be preferentially expressed in neonatal transitional B-1a progenitors (CD19^+^CD93^+^B220^lo/-^CD5^+^) and adult B-1 cells (CD19^+^CD43^+^B220^lo/-^) in B-cell lineage, we, in this study for the first time, identified another population of neonatal Bhlhe41-expressing B cells that were CD19^hi^dTomato^+^B220^hi^CD43^lo^CD5^lo^ at the stage of T1 and T2. On one hand, this may explain high *Bhlhe41* expression in transitional B cells observed in the public gene expression microarray data. On the other hand, the expression of CD5 and higher CD19 expression suggests CD19^hi^dTomato^+^B220^hi^CD43^lo^CD5^lo^ B-cell population as a potential novel population of splenic transitional B-1 progenitors.

It has been established that more than 90% of splenic B cells are immature transitional B cells expressing CD93 in neonatal mice within 1 week after birth (11, 21). Although we observed a lower expression of CD93 on this novel population of B cells, we demonstrated that CD19^hi^dTomato^+^B220^hi^CD43^lo^CD5^lo^ cells gave rise to both peritoneal B-1a and B-1b cells by adoptive transfer experiment. Bhlhe41 deficiency reduces neonatal splenic transitional B-1a progenitors (10). Using Bhlhe41^dTomato-Cre/dTomato-Cre^ mice as *Bhlhe41*-deficient mice, we also observed decreased splenic transitional B-1a progenitors upon loss of *Bhlhe41*. However, we surprisingly identified that *Bhlhe41* deficiency led to increased dTomato^+^ B cells, which was ascribed to a significant increase in CD19^+^dTomato^+^B220^hi^CD5^lo^ B cells. An altered balance between CD19^+^dTomato^+^B220^hi^CD5^lo^ and transitional B-1a progenitors (CD19^+^CD93^+^ B220^lo^CD5^+^) upon loss of *Bhlhe41* indicates that Bhlhe41 is functional in regulating both populations. We speculate that the two populations may have a developmental connection, which is worthy to be further studied. Of note, the characterization of B-1 cells and B-1 progenitors in this study was based on flow cytometric analysis of B-1 cell–associated surface markers. Further consolidation of this finding is warranted by analyzing immunoglobulin gene rearrangements.

In line with preferential Bhlhe41 expression in neonatal and adult B-1 cells rather than B-2 cells, we demonstrated a robust deletion of floxed genes in neonatal B-1 progenitors and peripheral B-1 cells. Despite less than 1% of B-2 cells expressing Bhlhe41, we did find DNA recombination of floxed genes in approximate 15% of B-2 cells from the spleen and bone marrow of *Bhlhe41^dTomato-Cre/^* ^+^ *Rosa26^EYFP^* mice. A similar level of DNA recombination was also observed in other major immune cells. This suggests that Bhlhe41 may be expressed in certain hematopoietic progenitors. We have excluded this possibility by showing that dTomato was not observed in neonatal bone marrow cells with enriched hematopoietic progenitors (data not shown), which is in agreement with the undetected *Bhlhe41* transcript in hematopoietic progenitors according to the gene expression microarray data by the Immunological Genome Project (16). However, it can also be due to transient Bhlhe41 expression in these cells or leaky expression.

As a robust DNA recombination was also observed in alveolar macrophage and microglia, utilization of *Bhlhe41^dTomato-Cre^* mouse for targeting the gene of interest in B-1 cells should be carefully considered when studying innate immune responses. We propose that developing an inducible Cre-ERT system under the control by *Bhlhe41* locus may improve the specificity of *Bhlhe41* promoter-driven DNA recombination in B-1 cells, alveolar macrophage, and microglia. Moreover, developing such a transgenic mouse may also be beneficial for fate-mapping B-1 cell development by crossbreeding with *Rosa26^EYFP^* mice.

Previous studies show that Bhlhe41 is preferentially expressed in B-1 cells, alveolar macrophage, microglia, *in vitro* differentiated Th2 cells, and activated follicular B cells in immune system (10, 14, 15). Using *Bhlhe41^dTomato-Cre/^* ^+^ *Rosa26^EYFP^* mice, we failed to observe Bhlhe41 expression in *in vitro* differentiated Th2 cells and activated follicular B cells. This discrepancy may be due to heterozygous deletion of *Bhlhe41* in *Bhlhe41^dTomato-Cre/+^* mice, or the limitations of techniques used in previous studies, e.g., false positive due to real-time PCR analysis of *Bhlhe41* in Th2 cells and unspecific staining by anti-human CD2 on activated follicular B cells from *Bhlhe41-Cre-hCD2* reporter mice (10, 15).

In summary, we identified neonatal CD19^hi^dTomato^+^B220^hi^CD43^lo^CD5^lo^ cells as novel transitional B-1 progenitors. *Bhlhe41* ^dTomato-Cre/+^ mouse can be used for fate mapping and functional studies of B-1 cells in host immune responses.

## Data availability statement

The original contributions presented in the study are included in the article/**Supplementary Material**. Further inquiries can be directed to the corresponding authors.

## Ethics statement

This study was reviewed and approved by The Animal Experimental Ethics Committee of Xuzhou Medical University.

## Author contributions

HL, YT, JR, RB, LaH, WJ, YC, LiH, MX, SG, YS and SP performed the experiments. HL, YT and HW performed data analysis and prepared the figures. HW contributed to conception and design of the study and manuscript writing with input by HL, SZ, QZ and LW. All authors contributed to the article and approved the submitted version.

## Funding

This work was supported by the National Natural Science Foundation of China (grant nos. 82171791 and 81872114), Jiangsu Provincial Special Program of Medical Science (grant no. BE2019617), Xuzhou Technology Program (grant no. KC20089), the Youth Innovation Team Grant and the Starting Grant by Xuzhou Medical University (grant no. D2018009), and the Postgraduate Research and Practice Innovation Program of Jiangsu Province, China (KYCX22_2875, KYCX21_2638, and KYCX22_2913).

## Conflict of interest

The authors declare that the research was conducted in the absence of any commercial or financial relationships that could be construed as a potential conflict of interest.

## Publisher’s note

All claims expressed in this article are solely those of the authors and do not necessarily represent those of their affiliated organizations, or those of the publisher, the editors and the reviewers. Any product that may be evaluated in this article, or claim that may be made by its manufacturer, is not guaranteed or endorsed by the publisher.

## Supplementary material

Supplementary Figure 1 Expression of *Bhlhe41* in immune cells. The public gene expression microarray data (GSE15907) were quantile normalized and *Bhlhe41* expression in murine immune cells was shown with arbitrary unit. LT, long term (LT); ST, short term; HSC, hematopoietic stem cell; MLP, multi-lymphoid progenitor, MDP, monocyte-dendritic cell progenitor; GMP, granulocyte-macrophage progenitor; MEP, megakaryocyte-erythrocyte progenitor; CLP, common lymphoid progenitor; CDP, common dendritic cell progenitor; DN, double negative; DP, double positive; BM, bone marrow; FL, fetal liver; ETP, early thymic progenitor; SP, single positive; NT, naïve T cell; MT, memory T cell; pDC, plasmacytoid dendritic cell; cDC, conventional dendritic cells. NKT, natural killer T lymphocyte; NK, natural killer cell.

Supplementary Figure 2 DNA sequencing of *Bhlhe41* locus in *Bhlhe41^dTomato-Cre^* mouse. The upper panel and lower panel show DNA sequence of the 5’ arm and 3’arm of transgenic *Bhlhe41* locus in *Bhlhe41^dTomato-Cre^* mouse, respectively.

Supplementary Figure 3 Expression of Bhlhe41 in tissues. **(A)** Expression of Bhlhe41 (dTomato^+^, green) in various tissues was examined by immunofluorescence analysis. **(B)** Analysis of Bhlhe41 expression (dTomato^+^, green) in microglia (Iba-1^+^, red) and alveolar macrophage (CD68^+^, red).

Supplementary Figure 4 Bhlhe41 is expressed in neonatal splenic CD19^hi^cKit^+^Sca1^+^ B-1 progenitors. Splenic B cells from neonatal *Bhlhe41^+/+^* and *Bhlhe41^dTomato-Cre/+^* mice at postnatal day 7 were analyzed by flow cytometric analysis.

Supplementary Figure 5 Cre-mediated DNA recombination in alveolar macrophage, microglia, and thymocytes by *Bhlhe41^dTomato-Cre^* mice. Flow cytometric analysis dTomato and EYFP in **(A)** alveolar macrophage, **(B)** microglia, and **(C)** thymocytes from adult *Bhlhe41^+/^* ^+^ and *Bhlhe41^dTomato-Cre/^* ^+^ *Rosa26^EYFP^* mice. The gating strategy for each type is illustrated on the left panel. DN, double negative; DP, double positive; SP, single positive; BALF, bronchoalveolar lavage fluid; CNS, central nervous system.

Supplementary Figure 6 Purity of sorted CD19^+^dTomato^+^ B cells from neonatal *Bhlhe41^dTomato-Cre^ mice*. Splenic CD19^+^dTomato^+^B220^hi^CD43^lo^ and CD19^+^ dTomato^+^B220^lo^CD43^hi^ B cells were purified from neonatal *Bhlhe41^dTomato-Cre^* mice at postnatal day 7 by flow cytometric sorting. The purity of sorted cells was analyzed by flow cytometric analysis.
