# Supplementary figures and images for: Identification of novel B-1 transitional progenitors by B-1 lymphocyte fate-mapping transgenic mouse model Bhlhe41 dTomato-Cre
Source: Front Immunol. 2022 Sep 15;13:946202. doi: 10.3389/fimmu.2022.946202 (PMC9520467; doi:10.3389/fimmu.2022.946202)

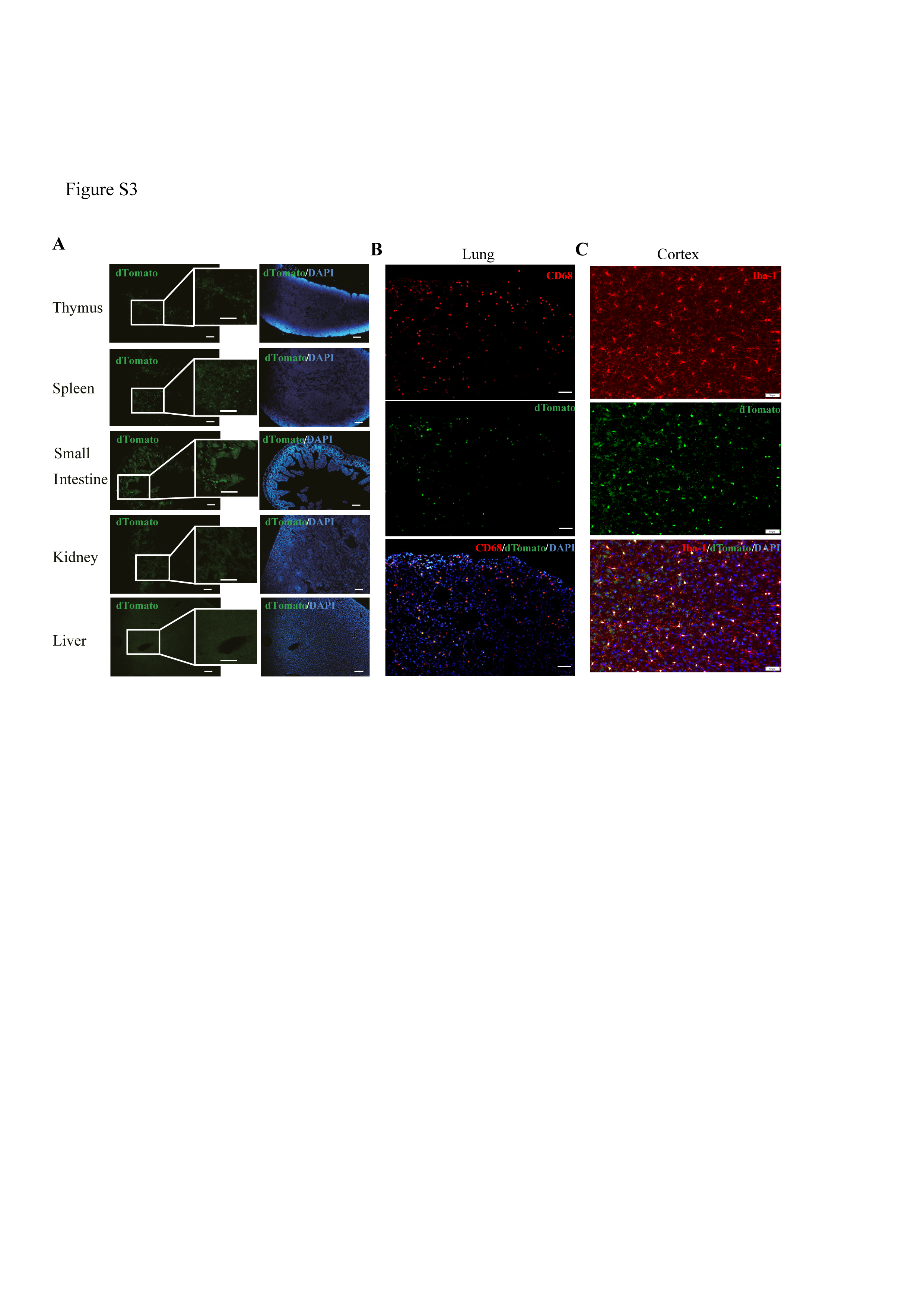

Supplement: Supplementary Figure 3 — Expression of Bhlhe41 in tissues. (A) Expression of Bhlhe41 (dTomato+, green) in various tissues was examined by immunofluorescence analysis. (B) Analysis of Bhlhe41 expression (dTomato+, green) in microglia (Iba-1+, red) and alveolar macrophage (CD68+, red). [file Image_3.tif]
